# Supplementary material for: Are carbon emissions trading and green financial instruments synergistic? -Comprehensive quantitative research based on content analysis
Source: PLoS One. 2024 Mar 7;19(3):e0298601. doi: 10.1371/journal.pone.0298601 (PMC10919601; doi:10.1371/journal.pone.0298601)
Supplement: S1 Data — (ZIP) [file pone.0298601.s001.zip › Data/地级市Z_F工作报告.zip!/▒▒╛⌐/└┤╫╘╣1⁄2╓┌║┼í╛┬φ┐╦╩2╛▌═°í┐_2007.docx]

各位代表：

　　现在，我代表北京市人民政府，向大会作政府工作报告，请予审议，并请市政协各位委员提出意见。

　　2006年工作回顾

　　过去的一年，在中共北京市委的领导下，首都人民坚持以邓小平理论和“三个代表”重要思想为指导，全面落实科学发展观，紧紧围绕“新北京、新奥运”战略构想，加快结构调整和增长方式转变，加强公共服务和社会管理，着力推进奥运筹备和城乡建设，经济社会持续稳定较快发展，构建和谐社会首善之区取得新的成绩。市十二届人大四次会议确定的主要任务胜利完成，实现了“十一五”时期的良好开局。

　　——初步统计，全市实现地区生产总值7720.3亿元，比上年增长12%，人均地区生产总值超过6000美元。一、二、三产业比重分别为1.3%、28.7%和70%。地方财政收入1117.2亿元，增长21.5%。居民消费价格指数为100.9%。万元地区生产总值水耗下降11.2%。

　　——城市居民人均可支配收入19978元，实际增长12.2%；农民人均纯收入8620元，实际增长8.7%。城镇登记失业率1.98%。

　　——市区空气质量二级以上天数241天，占全年的66%；化学需氧量和二氧化硫排放量分别下降5.2%和7.9%；城八区和郊区污水处理率分别达到90%和42%，城八区和郊区生活垃圾无害化处理率分别达到96.5%和57.5%；全市林木绿化率达到51%。

　　——为市民办的59件重要实事全面落实。

　　一、经济发展保持良好态势

　　认真落实中央宏观调控政策措施，严格清理固定资产投资项目，依法加强规划审批和土地管理，全社会固定资产投资增长19.3%。投资结构进一步改善，第三产业投资增长24.5%，房地产开发投资比重下降2.9百分点。制定了住房建设规划，落实“两限两竞”政策，经济适用住房供地增长80.3%，竣工270万平方米。消费需求稳步提升，住房、汽车、文化、旅游消费持续攀升，社会消费品零售总额增长12.8%。

　　产业结构高端化发展趋势更加明显。制定文化创意产业促进政策，产业集聚区初具规模；金融业快速发展，一批著名金融机构入驻北京；现代服务业增长12.8%，占地区生产总值的47.1%。工业结构调整稳步推进，高技术制造业对工业增长的贡献率达49.2%。都市型现代农业发展水平继续提升，完成设施农业建设4.5万亩，乡村旅游业快速发展。

　　转变增长方式迈出新步伐。大力发展循环经济，工业等重点产业节能降耗成效明显。新增节水灌溉农田30万亩。首钢调整、焦化厂搬迁顺利推进，煤矿和非煤矿山整顿关闭取得新成效。加强经济运行调节，煤电油气运等保障水平不断提高。

　　二、新农村建设扎实起步

　　制定了新农村建设实施意见和配套政策，完善了“部门联动、政策集成、资金聚焦、资源整合”的机制，实施108项新农村建设折子工程，80个市级试点村建设稳步推进。基础设施建设和社会事业发展的重点逐步向农村倾斜，市政府对农村投入达到111.8亿元。基本完成集体土地地籍和土地利用现状调查。“农村亮起来、农民暖起来、农业资源循环起来”工程建设全面展开，推广利用太阳能、沼气等新能源，搭建节能卫生吊炕，六环路以外40%的村庄环境整治任务全面完成，80%的平原地区实现垃圾密闭化管理，农村改厕11万户。完成自然村通油路471公里，解决了32万农民饮水安全问题。加强农村物流配送体系建设，新建和改造10个配送中心，发展连锁超市、便利店1623家。完善“五河十路”绿色产业带政策。山区小流域综合治理、废弃矿山生态修复、移民搬迁加快推进。通过开发整理，新增耕地4万亩。创新农村管水、乡村公路养护机制，为农民开发出近1.5万个公共服务就业岗位。招聘2000多名大学毕业生担任“村官”。防控禽流感疫情、美国白蛾侵害取得阶段性成果。农村各项改革稳步推进，社会事业发展全面提速，民主管理制度进一步完善。

　　三、奥运筹办推动城市建设与管理迈出新步伐

　　按照“安全、质量、工期、功能、成本”五统一的要求，奥运工程建设顺利推进。52个奥运场馆及相关设施如期开工建设，12个新建场馆和5个相关设施主体结构全部完工，国家体育场钢结构、国家游泳中心膜结构安装完成，丰台垒球场成功举行测试赛。深化中心城控制性详细规划编制，11个新城规划全部完成。5条轨道交通建设进展顺利，305公里高速公路、159公里城市快速路和主干路开工建设，京承二期、机场北线、通惠河北路等建成通车。优化调整131条公交线路，开通48条小区公交线路，全面推行市政交通一卡通，制定并启动优先发展公共交通方案，实施公交低票价政策。加快建设垃圾、污水处理等一批环境基础设施。稳步推进文保区试点和危旧房改造，实现解危排险9372户，完成城八区平房院水表改造7万户。一批文物建筑得到修缮保护。环境综合整治全面展开，拆除违法建设450万平方米，自拆比重95.4%，实现绿化130万平方米，80个“城中村”以及重点大街、重点地区、航空走廊整治取得明显成效。建立应急管理体制，全面排查、整改地下管网等各类安全隐患，向市民免费发放600万本防灾应急手册。集中整治“黑车”、小广告、违规养犬等突出问题。发挥市民主体作用，创新城市管理方式，开通信息化城市管理系统，建立无缝隙管理责任制，利用多种手段加强信息沟通，城市运行服务管理能力明显提高，在烟花爆竹“禁改限”、中非合作论坛服务保障、西直门立交桥改造等工作中初步得到体现。

　　四、市场监管力度稳步加大

　　围绕群众反映强烈的重点领域和产品，整顿和规范市场秩序。加强食品安全管理，建立信用公示和追溯体系，对21家企业、1480种不合格食品实行强制退市，强化对食品加工企业、小作坊的分类监管，严厉打击生产销售假冒伪劣食品的违法行为，食品抽查合格率达95.3%。严格药品认证和准入制度，坚决治理药品虚假广告，积极应对“齐二药”、“欣弗”事件，药品抽验合格率达98.6%。加强知识产权保护，在小商品市场和服装市场推行商标授权经营制度，严厉打击侵权盗版和商业欺诈行为，查处一批有较大影响的案件。推进企业信用体系建设，加强信息归集、整理、公示及应用，引导企业诚信经营。对存在安全隐患、污染扰民的企业进行治理，依法关闭和撤销汽配、建材、小商品等各类市场79家。强化房地产市场监管，对经济适用住房的建设和交易实施全过程监控，整治和查处房屋交易中的违法违规行为。对工程建设、土地出让、医药购销等15个重点领域进行整顿，切实规范市场主体行为，维护良好的市场秩序。

　　五、公共服务水平进一步提高

　　完善了15项促进就业配套政策，加大对就业困难群体和地区的帮扶力度，转变残疾人就业保障金征缴方式，新增城镇就业人员34.4万人，城镇登记失业人员实现就业19.1万人。88%的行政村建立了就业服务站，培训农村劳动力10.8万人次，实现转移就业8.7万人。制定北京市基本养老保险规定，解决了建立企业年金单位缴费的税前列支问题，区县出台了农村社会养老保险改革办法。企业退休人员统一补充医疗保险办法开始实施，新型农村合作医疗参合率达到86.9%。自收自支和部分补助事业单位、民间非营利组织纳入工伤保险范围。参加工伤、医疗保险的农民工分别达到120.3万人和114.5万人。建立农村最低生活保障标准调整机制，提高城市居民社会保障相关待遇标准和特殊困难人员的救助标准，扩大廉租房救助范围。社会保险基金运行平稳。

　　启动社区卫生服务收支两条线管理，对常用药实行集中采购、统一配送和零差率销售，完成农村532个社区卫生服务机构标准化建设；在二级以上医院设置惠民病床，三级医院部分检验结果实行互认通用，公共卫生体系建设取得初步成果。积极推进义务教育均衡发展，全部农村和城镇低保家庭学生享受“两免一补”政策，实施中小学办学条件标准化建设，选派1105名城镇中小学教师到农村支教，培训3000名农村中小学骨干教师。加强职业教育统筹规划，高等教育办学质量不断提高。创新公共文化服务模式，进行社区、乡镇文化活动中心标准化建设，推出农村“文艺演出星火工程”和区县“周末演出计划”。基本实现农村广播电视村村通、户户通。公布了首批非物质文化遗产名录。文化体制改革顺利推进，文化市场更加繁荣。促进群众体育与竞技体育协调发展，改善社区和行政村全民健身设施，成功举办一系列重大赛事，北京籍运动员在第十五届亚运会上获得42枚奖牌。

　　六、改革开放和科技创新扎实推进

　　实施投资项目备案制和核准制，规范固定资产投资项目办理流程，试行政府投资项目储备、后评价和重大项目公示制度。国有企业改革取得新进展，基本完成地方国有控股上市公司股权分置改革和一级企业5个重组项目，全面完成清产核资工作，初步搭建起国有资本预算管理制度框架。金融改革迈出新步伐，市属证券公司完成重组并实施清算。清理限制规定，制定鼓励政策，引导和支持非公有制经济发展。推进资源性产品价格改革，调整非民用天然气销售价格，建立出租汽车油价与租价联动机制。按照城乡统筹的要求，改革园林绿化管理体制。出台促进行业协会发展和改革的意见，积极推进政会分开。电子政务建设取得重大进展，城市管理、公共服务等领域信息化水平不断提高。

　　对外开放积极活跃。实际利用外资45.5亿美元，增长29.1%，第三产业吸收外资比重不断提高。地区商品进出口总额增长26%，服务贸易保持快速增长。奥运对旅游业发展的促进作用日益显现，全年接待入境游客390.3万人次，增长7.5%。对外合作交流更加深入，新增6个国际友好城市，成功举办国际文化创意产业博览会、诺贝尔奖获得者北京论坛、中俄国家年“莫斯科周”等大型活动。与河北省签订合作协议，京津冀区域协调发展得到加强。

　　加快落实全国和市科技大会精神，全面启动创新型城市建设，出台了实施意见及配套政策措施。促进企业创新和成果转化，支持63家科技专业中介组织为企业服务，统筹使用科技经费，集中资金2.4亿元支持科技成果转化。中关村科技园空间布局不断优化，非上市股份公司股份报价转让、创业投资发展等体制创新取得突破，支持清华、北大及中科院等单位的8个实验室对企业开放。在集成电路、疫苗、新能源、生物农业技术等领域取得了一批自主创新成果。科技交流和科学普及工作深入开展。全年专利申请2.7万件，实现技术交易额572.6亿元。

　　七、精神文明与民主法制建设得到加强

　　深入学习邓小平理论和“三个代表”重要思想，认真组织开展《江泽民文选》学习。大力弘扬以“八荣八耻”为主要内容的社会主义荣辱观，“迎奥运、讲文明、树新风”活动广泛开展，精神文明创建活动蔚然成风，未成年人思想道德建设和大学生思想政治教育得到加强。积极倡导文明办网、文明上网，网络环境得到改善。国防教育和后备力量建设扎实推进，军政、军民关系更加密切。妇女、儿童、老年人、残疾人事业健康发展。民族、宗教、侨务和对台工作取得新进展。

　　认真执行市人大及其常委会决议，重大决策广泛征求市政协和各民主党派的意见。自觉接受各方面监督，充分听取广大群众对政府工作的意见和建议。市政府组成人员认真向市人大常委会报告专项工作，积极参加市政协议政会。办理全国和市人大代表议案5项、建议1762件，全国和市政协委员建议案9件、提案1284件。启动奥运立法工作。全面推行行政执法责任制，进一步规范执法行为。普法宣传工作力度进一步加大，社区矫正工作机制更趋完善。基层民主管理和村民自治得到加强，和谐社区、和谐村镇建设稳步推进。认真开展治理商业贿赂专项工作，廉政建设取得成效。强化社会治安防控体系建设，社会治安秩序进一步改善。

　　各位代表！一年来所取得的成绩，是党中央、国务院和市委正确领导的结果，是全市各族人民团结奋斗、开拓进取的结果。在这里，我代表市人民政府，向全市各族人民，向人大代表、政协委员以及社会各界人士，向中央各部门、兄弟省区市和驻京部队，向所有关心和支持首都建设的香港与澳门特别行政区同胞、台湾同胞、海外侨胞和国际友人，表示崇高的敬意和衷心的感谢！

　　回顾总结工作，我们也清醒地看到，经济社会发展还存在诸多矛盾和问题，人口资源环境矛盾尚未得到缓解，尤其是人口规模增长过快，治理环境污染、交通拥堵的任务非常艰巨，城市管理水平亟待提高；经济增长方式还比较粗放，城乡、区域差距较大；安全生产形势依然严峻；保障性住房供应不足，商品房市场结构不合理，价格上涨过快，违法违规用地屡禁不止；就业、社会保障、收入分配、教育、医疗、食品药品安全等关系群众切身利益的问题还需下大力气解决。特别是政府职能转变还不到位，一些政府部门及其工作人员服务意识不强，极少数干部以权谋私、严重腐化堕落。对上述问题，我们将高度重视，迎难而上，采取切实措施加以解决。

　　2007年工作安排

　　综观国内外形势，首都发展既面临着良好机遇，也面对着严峻挑战。2007年是奥运筹备决战之年，今天距奥运会开幕仅剩560天，完成好全年各项工作，时间紧、任务重、责任大，容不得半点松懈。我们要以奥运筹备带动全市各项工作的开展，切实增强紧迫感、责任感、使命感，紧紧抓住机遇，敢于应对挑战，努力化解风险，全市动员、全力以赴、全面完成首都发展建设的各项任务，不断提升城市现代化水平，不断提升城市文明程度，不断展示首都新形象。

　　2007年政府工作的总体要求是：以邓小平理论和“三个代表”重要思想为指导，认真贯彻党的十六大和十六届三中、四中、五中、六中全会及中央经济工作会议、胡锦涛总书记考察奥运会工程建设时的重要讲话精神，全面贯彻落实科学发展观，加快构建社会主义和谐社会首善之区，紧紧围绕“新北京、新奥运”战略构想，切实推动经济结构调整和增长方式转变，扎实推进社会主义新农村建设，努力提高城乡建设服务管理水平，深入推进改革开放和自主创新，大力发展各项社会事业，全力完成奥运会的筹备任务，以首都经济又好又快发展、社会和谐稳定的良好局面迎接市第十次党代会和党的十七大胜利召开。

　　全市经济社会发展主要目标是：地区生产总值增长9%，万元地区生产总值能耗、水耗分别下降5%，市区空气质量二级以上天数达到67%，化学需氧量和二氧化硫排放量分别下降3%和10%；城镇登记失业率控制在2.5%以内，居民消费价格指数调控在102%左右；地方财政收入增长13%，城乡居民收入实际增长7%。为群众办好58件重要实事。

　　一、举全市之力做好奥运会、残奥会筹备工作，着力提升城市建设服务管理水平

　　认真落实奥运三大理念，加快实施“倒排期”工程，扎实推进奥运场馆和基础设施建设，更加注重生态环境、市容环境、运行环境和社会环境建设，全面提升城市管理和服务水平。

　　(一)切实完成奥运场馆和城市基础设施建设任务。

　　2007年，除国家体育场外，30个奥运会比赛场馆和45个训练场馆要全部完工，5个相关设施和场馆周边道路建设基本完成，外围市政工程、景观工程、绿化工程建设全面展开。严格落实“五统一”量化指标考核体系，强化工程业主及参建各方的主体责任，突出工期控制，确保工程安全质量。加强与奥组委的沟通，做好工程建设与使用功能、场馆运行管理的有效衔接和整体推进。按照绿色环保、简洁实用、精心组织、协同作业的要求，实施场馆装修和机电设备安装，高质量完成后期建设，努力把奥运场馆建设成精品工程。加强审计监督，实施“阳光工程”。

　　贯彻落实城市总体规划，强化城市公共服务设施、基础设施和公共安全配套设施的规划建设，提高规划监管力度。全力推进轨道交通建设，新开工地铁8号线二期、9号线、10号线二期、轻轨亦庄线，实现地铁5号线通车试运营。建成朝阳路、安立路大容量快速公交线路。加快6条高速公路、3条城市快速路、17条城市主干路建设，完善换乘枢纽和场站设施，大力支持停车设施建设，改善城市道路微循环系统和非机动车及步行环境，加快推进城市无障碍设施建设。新建首都图书馆二期等工程，首都机场扩建工程等项目竣工，国家大剧院7月投入使用。发展城市管网集中供热300万平方米。加快南水北调北京段工程建设，建设吴家村等3个再生水厂。完善相关政策，积极治理非正规垃圾场，加快生活垃圾焚烧厂建设，新建和改建公厕千余座。

　　(二)着力推进奥运环境建设。

　　切实改善生态环境。实施第十三阶段控制大气污染措施，完成中心城区20蒸吨以下燃煤供暖锅炉改造，加快淘汰老旧高排放车辆，开展油气回收治理，严格控制扬尘污染。限期治理水源保护区内的污染源，完成六环路以内城市河湖治理，加快污水和再生水管网建设，加强排水设施安全运行管理，提高再生水水质。推广先进技术，建立生活垃圾处理经济补偿机制，加快垃圾减量化设施建设，提高垃圾处理资源化、无害化水平，城八区、郊区生活垃圾无害化处理率分别达到97%和60%。加强对施工噪声、社会生活噪声的查处，对噪声扰民严重的交通路段进行治理。强化危险废弃物、放射性废物和放射源的监管。认真做好污染源普查和土壤污染调查。继续开展三道绿色生态屏障和城市大绿地建设，全市林木绿化率达到51.6%。

　　全面整治市容环境。加强奥运比赛场馆、定点饭店周边及途经道路沿线的治理和建设。全面开展城乡结合部地区环境整治，以政府为主导、城乡居民为主体，坚持制度创新，加大公共财政投入，完善管理体制，切实解决环境脏乱差、发展不协调等问题。完成22个“城中村”和城市边角地、老旧小区、航空走廊、铁路沿线环境整治任务，拆除违法建设332万平方米。落实重点大街、重点地区环境建设规划，实施楼房平改坡工程，清洗粉饰建筑物外立面，做好胡同街巷整治，加快规范公共场所英语标识。围绕治安、交通、市场、环卫、旅游等领域，建立完善奥运期间城市运行指挥体系和管理模式，继续整治“黑车”、小广告、违规养犬等突出问题。

　　不断优化城市运行环境。认真做好23项测试赛工作，建立场馆外围保障工作机制。确定奥运赛时城市运行“体征指标”体系，搭建奥运城市运行指挥控制平台，实现奥运筹办工作的重心和体制、机制向奥运赛时转变。推动应急组织管理体系、预案体系和工作运行机制向基层延伸，大力普及公共安全、生产安全和应急知识，开展综合应急演练。加强各类突发公共事件风险隐患的排查和监控，重点消除电网、地下管网、道路桥梁存在的安全隐患。健全安全生产综合监管、行业监管、属地监管体制机制，严格实施人员密集场所安全规定，继续开展高危行业安全专项治理，加大安全生产监督检查执法力度。按照“公交主导、体制改革、线路调整、管理加强”的思路，落实优先发展公交措施，优化线网，扩大路权优先，加强静态交通管理，着力缓解城市交通拥堵。全力推进城市运行纲要的实施，确保117项城市运行项目的落实。

　　努力营造良好的社会环境。全面实施人文奥运行动计划，深入开展“迎奥运、讲文明、树新风”活动，切实提升市民文明素质和城市文明形象。进一步抓好文明礼仪教育和市民讲外语活动，广泛开展奥运知识宣传教育。加强商业、餐饮、旅游、公共交通等窗口行业奥运培训，提高从业人员的业务技能和服务水平。加强公共场所秩序建设，着力培养广大市民守规章、守秩序的文明习惯。加快推进奥运立法工作，制定奥运期间城市运行的相关规章制度。积极配合奥组委做好奥运会、残奥会志愿者的招募和培训工作。动员社会各界力量，采取多种形式，解决好影响首都形象的突出问题，营造文明、热情、友好、健康的社会氛围。

　　二、加快结构调整和增长方式转变，推动首都经济又好又快发展

　　坚持以节约能源资源和保护环境为切入点，严把土地供应、规划审批和市场准入门槛，着力调整经济结构和转变增长方式，努力实现科学发展。

　　(一)认真落实中央宏观调控各项政策措施。

　　合理控制固定资产投资规模，清理国家严令限制的一般加工项目，保障奥运工程、基础设施、新农村建设、结构调整等方面投资。严格控制耕地转为建设用地规模，积极推进实施工业用地“招拍挂”出让，坚决查处各种违法违规用地行为。国有土地使用权出让收支纳入地方基金预算管理，优化土地出让金使用结构。把握好土地供应总量和开发建设规模，调整房地产结构和布局，保持房地产投资平稳增长。推进实施住房建设规划，增加中小户型、中低价位住宅建设用地，积极促进二手房交易市场发展，抑制房价过快增长。完善经济适用住房管理办法，出台政策性租赁住房政策，缓解低收入家庭的住房困难。努力提高农民和城镇中低收入家庭的收入水平，密切监测群众生活必需品市场供求和价格变动，稳定消费预期，增强消费需求的拉动作用。全面落实商业服务业迎奥运三年行动计划，推进奥运餐饮服务、特色商业街区建设等工作，营造安全、规范、便利的消费环境。健全经济运行各项调控预案，建立联合调度协调机制，做好煤电油气运保障工作。

　　(二)加大产业结构调整力度。

　　落实促进文化创意产业发展的各项政策，制定文化创意产业发展规划。安排专项发展资金，支持重点产品、服务和项目，加快文化创意产业集聚区基础设施建设，有序引导各区县发展有特色有优势的项目。抓好北京出版社出版集团等单位改革，稳步推进经营性文化事业单位改制。制定促进体育产业发展的意见，鼓励社会资本和外资投资体育产业，加快培育体育品牌企业集团和大型体育赛事。抓住银行业全面开放的机遇，落实鼓励金融业发展政策，加快金融机构后台服务支持体系建设，吸引国内外金融机构在北京发展。促进信息服务业、商务服务业加快发展。建设奥运旅游大道，扶持乡村旅游基础设施建设，大力开发旅游演出和旅游商品。加快电子信息、生物医药等高技术产业发展。支持重大精密技术装备制造项目引进和自主研发，继续推进首钢冷轧、中芯国际增资扩产、康宁玻璃基板生产线等项目。落实北京名牌产品建设有关措施，在技改项目、研发投入和升级改造方面给予定向支持。加快建设服务绿色通道，加强对中央企事业单位联络服务工作。

　　(三)加快功能区建设。

　　落实区县功能定位，研究制定具体政策，继续加大对山区、南城的支持力度，统筹推进各功能区的协调发展。全面落实支持中关村科技园区发展的各项政策，加快国家工程技术创新基地、软件产业基地、生物医药产业基地建设，逐步形成创新型产业集群。加强北京经济技术开发区交通和能源等基础设施建设。加快金融街、商务中心区重大项目建设，积极发展配套服务产业。促进临空经济区、奥林匹克中心区内部资源整合。高度重视高端产业功能区的管理体制创新，建立健全统筹协调机制，积极引入高端企业和高级人才，提高专业化服务水平，促进高端产业功能区健康发展。

　　(四)积极发展循环经济。

　　大力推进节能降耗减排。坚决退出高耗能、高污染产业，重点抓好首钢压产和焦化厂、化工二厂、有机化工厂的搬迁工作，继续清理整顿煤矿和“五小”企业，关闭80个固体矿山，加大产业转型扶持力度。建设循环经济示范园区，支持石化、建材、电力等重点行业开展清洁生产，从源头上削减废弃物的产生量。加强能源统计、分类计量和能耗信息监测平台建设，完善分区域、分行业、重点企业节能降耗的监督考核体系。继续做好大型公建节能，新增居住建筑达到节能65%的设计标准，推进非节能建筑的改造，推广绿色照明和节能新技术，政府机构带头节能、节水、使用再生纸，努力建设节约型社会。强化计划用水定额管理，对农业用水试行征收水资源费，建设雨水收集设施，利用再生水4.8亿吨。实施产业用地定额指标和土地投资强度指标管理，集约利用开发区土地。开展再生资源回收产业化试点，规范社区回收网络，建设专业化分拣中心，支持再生资源回收利用企业发展。

　　(五)进一步强化市场监管。

　　严格市场准入制度，对不合格产品坚决实行强制退市。严格市场设立标准，限制低水平市场建设。健全市场信息公告、预警预报和快速反应机制，整合完善市场监管综合调度指挥系统，建立市场秩序综合评价指数。以生产和销售环节为重点，完善食品安全监控和追溯体系，建立5000个监控点，对50大类、2500种食品进行全面监测，切实保证市民食品安全，抓好奥运食品安全保障工作。健全药品安全监测网络，建立上市药品安全再评价机制，在264家生产企业建立药品不良反应采集系统。加强房地产市场监管，重点打击囤房、炒房，遏制投机性购房。建立违法广告信息披露和市场退出机制，依法查处各种违法违规行为，切实维护市场经济秩序。加快社会信用体系建设，研究制定信息采集、使用管理办法和个人信用体系建设方案。

　　三、统筹城乡发展，推进新农村建设

　　进一步加大对“三农”工作支持力度，整合支农资金和资源，理顺部门联动关系，鼓励和支持社会力量参与新农村建设，逐步建立城市支持农村的长效机制。

　　(一)加快农村经济发展。

　　调整优化农村经济结构，加快都市型现代农业、农产品加工业、乡村旅游业、农村服务业发展，推动一产向二产、三产延伸。继续推进农业“七大体系”建设，抓好现代都市农业走廊和农业示范园区建设，切实提高农业综合生产能力。重点支持专业村、专业户发展，扶持龙头企业、农民合作组织和专业协会，促进农业产业化经营。鼓励农民自主创业，大力扶持农村个体私营经济，加快发展农村集体经济。做好无公害农产品、绿色食品和有机农产品认证工作，重点开发适合奥运期间市场需求的特色农产品。认真解决农产品运输、储藏和销售等方面的问题，保持农业生产资料价格稳定。开发休闲旅游线路，形成一批有地方特色、文化内涵的旅游产品。积极发展农村商业分销配送站点、连锁超市和便利店。继续推进小城镇建设，加快产业发展和聚集。

　　(二)加强农村基础设施建设。

　　深入推进80个市级试点村建设，带动新农村建设各项工作的开展。坚持抓好“亮起来、暖起来、循环起来”工程建设，在有条件的地区实施沼气、生物质气化集中供气工程。推广使用太阳能等清洁能源技术，加快农村地区燃气化进程。完成郊区公路三年提级改造任务，继续实施自然村通油路工程，支持村内道路硬化。大力发展农村客运，加快行政村通公交步伐，积极研究理顺体制票制方案。升级改造广播电视“村村通”系统，加强农村邮政基础设施和综合信息服务体系建设。完成30万农民安全饮水改造任务。扎实推进农村改厕工作，基本完成农村中小学厕所改造。

　　(三)全面推进郊区生态保护与环境建设。

　　落实“富民养山”政策，加强中幼林抚育，提高小流域综合治理的标准和质量，完成20条小流域综合治理，治理水土流失面积310平方公里。进行矿山生态破坏状况调查，编制生态修复规划，对1467公顷废弃矿山实施生态修复。制定实施生产生活条件恶劣地区移民搬迁政策，完成采空区、强泥石流易发区农户搬迁工程，研究妥善安置等后续政策。全面清理积存垃圾，加快农村排水和污水处理设施建设。着力推进农村环境整治，基本完成村庄整治任务，六环路以外95%的平原地区实现垃圾密闭化管理，创建环境优美乡镇和生态文明村。

　　(四)深化农村各项改革。

　　健全村庄规划建设管理体制，加强规划实施的监督管理。完善配套措施，健全农户土地承包经营权流转机制。制定集体土地征地留用安置办法，扩大集体建设用地流转试点范围，依法加强管理。建立公共服务设施管理和运行机制，组建村庄公共服务设施和环境管护队伍，做好日常维护，确保各项设施的正常运转。制定乡镇管理体制综合改革指导意见，选择若干乡镇进行试点。加快集体经济产权制度改革。推进农村金融产品服务创新，引导金融机构增加对“三农”的信贷投放，试行农业政策性保险制度方案。做好第二次全国农业普查工作。

　　四、坚定不移地推进改革开放，加快建设创新型城市

　　着力加强重点领域、关键环节的改革，提升对外开放水平，加快创新步伐，为首都各项事业发展提供不竭动力。

　　(一)深化行政管理体制改革。

　　优化和规范投资项目审批程序，建立科学便捷的投资项目管理机制。推进政府投资项目公示和后评价制度试点，严格实施项目评审考核制度，完善重点建设项目信息管理系统。推进国库集中收付制度改革，改革非税收入收缴管理制度，扩大集中收缴的范围和额度。加强部门预算一级单位的财务管理，提高财政资金使用效益。认真做好内外资企业所得税两税合并准备工作。制定事业单位总体改革思路及分类改革意见，继续深化民政事业单位管理体制、街道管理体制改革试点。制定促进行业协会发展和改革的配套政策，启动行业协会规范治理工作。推动城市管理综合执法体制改革，探索建立行政执法协调机制。加快电子政务建设，推进政务信息资源共享和行政许可事项网上协同办理。继续深化资源性产品价格改革，积极稳妥地进行供热体制改革。

　　(二)稳步推进国有企业改革。

　　健全国有资本预算管理制度，认真做好国有资本收益收缴和预算管理工作，加强对企业重大事项决策的监管。抓住当前国际国内资本市场发展的有利时机，加快竞争性领域国有企业重组改制上市步伐，积极引入战略投资者，拓展企业融资渠道，完善法人治理结构及运行机制。继续深化市属金融企业重组改革，防范和化解金融风险。加快推进劣势企业退出市场，妥善解决职工安置等遗留问题。进一步完善相关配套政策，支持非公有制经济健康发展。建立中小企业创业投资引导基金，开展创业投资试点。

　　(三)努力提高对外开放水平。

　　积极吸引跨国公司地区总部、研发中心和结算中心，争取更多国内行业龙头企业总部落户北京。在文化创意产业、高技术产业、金融业等重点领域，有针对性地开展专业化招商引资，取得对欧美地区投资促进工作的新进展。研究制定促进服务外包发展的政策措施。提高货物贸易、服务贸易增长质量，支持具有自主知识产权、自主品牌的商品出口。依托国家对外战略项目，积极推进企业开展海外投资。加强与周边省区市的联系与协调，深化水资源和能源利用、生态环境保护、基础设施建设等方面的合作。创新思路与机制，推动京津冀都市圈各类资源的合理配置，促进区域协调发展。进一步做好对口支援和帮扶工作。

　　(四)加快创新型城市建设。

　　发布中长期科学与技术发展规划，落实促进自主创新的各项政策措施，培育和引进自主创新人才，建立企业自主创新专项资金，引导企业设立研发机构，支持以企业为主体，以科研机构、高等院校为技术依托的产学研合作。完善科技资金投入机制，支持大学科技园、孵化器和科技中介机构发展，以项目为载体整合各类资源，形成支持企业创新的合力。健全知识产权应急处理和预警机制，建立知识产权公共信息服务平台。充分发挥中央在京科技资源的作用，争取国家重大科技项目落户北京。深化科技体制改革，积极实施科技主题计划，围绕解决城市发展中的难题，大力开展科技攻关和科技成果应用。制定农业技术推广体系改革方案，抓好10项农业技术试验示范项目，继续做好农业科技推广运用。落实全民科学素质建设工作方案。

　　进一步做强中关村科技园区，继续实施开放式实验室工程，抓好非上市股份公司股份报价转让、创业投资、中小企业信用制度等创新试点。开展中关村百家企业自主创新示范工作。建设“中关村国家标准化示范园”，支持企业标准联盟、技术联盟和产业联盟的发展，争取在软件和集成电路、移动通讯、生物医药、新能源等领域实现一批关键技术突破，创制一批先进技术标准。

　　五、努力提高公共服务水平，扎实推进和谐社会首善之区建设

　　从解决广大人民群众最关心、最直接、最现实的利益问题入手，以促进城乡统筹为目标，加快公共服务体系建设，提高社会管理水平，不断促进社会和谐。

　　(一)努力扩大就业。

　　切实落实各项促进就业政策，多渠道开发就业岗位，实现全市城镇新增就业33万人。强化对就业困难群体的政策扶持，提高用人单位招用失业人员的岗位补贴，放宽困难地区灵活就业人员补助年龄条件，扩大创业培训补贴的覆盖范围，促进17万城镇登记失业人员就业，实现“零就业家庭”中至少有1人就业。做好残疾人就业保障金的征缴和使用，促进残疾人就业。加强农村劳动力转移就业培训和服务，促进农民就业基地建设，健全城乡“手拉手”就业协作机制，完善农村就业困难人员岗位补贴政策，实现6万名农村劳动力转移就业。继续推进农村公益事业管理机制创新，为农民提供更多的公益性就业岗位。健全协调劳动关系三方机制，认真落实劳动合同和集体合同制度。强化对各类职介机构的监督检查，建设全市统一的劳动力市场信息系统。

　　(二)加快完善社会保障体系。

　　改革基本养老金计发办法，扩大个体工商户、灵活就业人员的覆盖面。落实农村社会养老保险实施办法，完善财政补贴政策。做好城镇在校学生、学龄前婴幼儿和城镇无保障老年人参加医疗保险试点工作。完善新型农村合作医疗筹资增长机制，逐步扩大参合农民受益面。将非参照公务员管理的事业单位纳入工伤保险范围。推行农民工工资专项支付制度，继续推进农民工参加医疗保险、工伤保险。加大扩面征缴和基金监管力度，确保基金安全。进一步完善城乡最低生活保障、分类救助、专项救助制度，继续提高城市居民社会保障相关待遇标准，大力推动社会福利、优抚、慈善事业发展。加大住房保障力度，新开工中低价位商品住宅300万平方米、经济适用住房200万平方米，建设、收购30万平方米廉租住房，对低保家庭住房困难户实现应保尽保。

　　(三)着力加强医疗卫生服务。

　　坚持公共医疗卫生的公益性质，采取多种措施，逐步解决看病难、看病贵问题。突出抓好以收支两条线为核心的社区卫生服务运行机制改革，建立药品集中采购、统一配送、零差率销售的长效机制，制定社区卫生服务管理制度和监督考核办法，发挥社区卫生服务“六位一体”功能。试行社区卫生服务首诊制，建立大医院与社区卫生服务机构之间的双向转诊机制。完成规划设置的所有农村社区卫生服务机构的标准化建设和基本设备配备，加强远郊区县区域医疗中心建设，完善农村药品供应网和监督网。进一步加强基层社区特别是农村卫生人才队伍建设，加大综合医院和专科医院对社区卫生服务机构的支援，实行专家巡诊制，注重对预防保健的业务指导和技术支持，切实提高社区医疗服务水平。推动医疗付费制度改革试点，推出二级以上医院部分检查结果互认等方便群众就医的新举措。加强医院管理，积极改善医患关系。继续推进公共卫生体系建设，完善传染病救治和医疗急救网络，进一步发挥中医药的独特作用，做好重大疾病防控工作。

　　(四)提高公共教育服务水平。

　　制定基础教育均衡发展的意见，提高公用经费定额标准，加大对财力薄弱区县义务教育经费的转移支付，推动城区义务教育免杂费工作。优化城乡中小学空间布局，加快中小学办学条件标准化建设。推进中小学人事和分配制度改革，探索城乡教师交流长效机制，促进优质教师资源合理流动。完善具体措施，依法保障农民工子女接受义务教育。开展“阳光体育运动”，提高中小学生身体素质。深化职业教育办学体制改革，试行双证书制度，抓好实训基地建设，建立资助体系，降低中等职业学校学费，推进农村职业教育、职工岗位培训和成人教育发展。坚持高等教育内涵式发展，加强管理，提高质量，办出特色。落实民办教育促进法实施办法，引导和规范民办教育发展。

　　(五)推进文化、体育事业发展。

　　加快社区和农村文化设施标准化建设，加强日常管理和维护。实施露天剧场建设办法，开展“体验中国”文化广场活动，培育“朝阳流行音乐周”等特色区县文化活动品牌，办好农民艺术节。抓好北京国际音乐节和戏剧、舞蹈演出季等品牌活动，推出一批具有国际水准的剧目。重视历史文化名城保护，采取有效措施对优秀近现代建筑进行保护，继续推进6片文保区试点工作。扩大有线电视数字化试点。组织实施“国家重大出版工程”等重点项目，举办好世界期刊大会等重大活动。全面启动第二轮地方志编修工作。加强公共体育设施规划建设，全市行政村健身设施配建率达到90%，促进学校体育设施向社会开放。积极开展体育文化系列活动，办好全民健身体育节、第六届农民运动会以及重大国际体育赛事。

　　(六)切实加强社会管理。

　　加强城乡基层自治组织建设，积极培育和发展社会组织，充分发挥居民的主体作用，维护居民的共同利益，深入推进和谐社区、和谐村镇建设。完善物业管理法规，健全物业服务标准和收费标准指导体系，强化属地管理，加大执法力度，妥善处理物业纠纷。成立流动人口和出租房屋管理委员会，加快区县、街乡相关管理机构建设，设立社区(村)管理站，修订流动人口户籍管理规定，搭建流动人口和出租房屋综合信息平台。积极探索调控人口规模的有效途径。落实《北京市信访条例》，做好人民建议征集工作。加强行政复议工作，推进人民调解的专业化、社会化建设，完善人民调解与行政调解、司法调解的衔接机制。健全社会治安防控体系，加快推进科技强警和信息化建设，依法严厉打击各种违法犯罪活动。落实各项工作措施，有效防范和严厉打击境内外敌对势力的破坏活动，强化反恐防爆工作机制，维护首都社会稳定。

　　(七)推进社会主义精神文明和民主法制建设。

　　认真搞好“三个代表”重要思想、科学发展观和构建社会主义和谐社会宣传教育活动，弘扬以爱国主义为核心的民族精神和以改革创新为核心的时代精神，深入实践社会主义荣辱观，建设社会主义核心价值体系，推动和谐文化建设。丰富和拓展精神文明创建活动的内涵，总结推广群众创造的新形式、新经验。加强未成年人思想道德建设基础工作，切实抓好大学生思想政治教育。加强网络文化的建设，规范网吧、互联网和手机短信管理，大力发展和传播健康向上的网络文化。坚持不懈开展“扫黄打非”工作。发挥哲学社会科学的重要作用，加强和谐社会重大理论问题研究。认真落实“五五”普法规划，加强和改进法律援助，建立健全农村法律服务体系。加强基层民主政治建设，继续完善政务、厂务、村务公开，做好第七届村民委员会换届选举工作。开展民族团结进步创建活动，加快少数民族乡村经济社会发展。贯彻党的宗教信仰自由政策，依法管理宗教事务。深入开展国防教育，支持驻京部队的现代化建设，加强国防后备力量建设，大力推进双拥共建。进一步做好外事、侨务、港澳台等工作。继续做好人口和计划生育工作，加强服务和管理。关心支持妇女、儿童事业，健全助残服务体系，积极探索养老服务模式，加强养老服务设施建设。

　　六、切实加强政府自身建设，努力建设服务型政府

　　加快政府职能转变，加强干部队伍作风建设，不断提高“四个服务”水平，努力做到“在服务中实施管理，在管理中体现服务”。

　　(一)切实加快政府职能转变。

　　履行经济调节、市场监管、社会管理、公共服务等四项职能，要求我们具体做好“规划、发展、建设、运行、服务、管理”等六个方面工作。“规划”就是编制并落实好城市总体规划、经济社会发展中长期规划，以及各种专项规划，强化实施监督检查，促进人口资源环境协调发展，促进经济社会可持续发展。“发展”就是创新体制机制，优化发展环境，着力推进结构调整和增长方式转变，解决好“三农”问题，大力发展社会事业，实现科学发展。“建设”就是完善城市功能与布局，搞好基础设施和公共服务设施建设，推进精神文明建设，提高城市的承载能力、现代化水平和文明程度。“运行”就是加强日常监管，消除安全隐患，及时处置各种突发事件，保证城市基础设施正常运转，保障市民生活必需品的正常供应，维护良好的市容环境。“服务”就是不断丰富“四个服务”的内涵，为各类市场主体服好务，为基层服好务，为市民提供全方位服务。“管理”就是坚持依法行政，不断转变管理观念，改革管理体制，创新管理方式。转变政府职能，既要充分发挥市场在资源配置中的基础性作用，又要切实承担起社会公益事业发展的责任。

　　(二)努力提高服务水平。

　　在城市管理中，市民是主体，政府负主责。各级政府、各部门要按照建设服务型政府的要求，强化服务意识，改进服务方式，提高服务质量和效率，为市民和基层提供方便快捷优质服务。对事关市民生产生活的工作，应做到“服务到家、管理到位”，寓管理于服务之中。只有服务到位了，管理才能到位；只有提高管理水平，才能提供优质服务。要认真分析服务对象，把握市民和基层需求，推动管理重心下移，在帮助他们解决面临的各种问题中实施管理。建立服务质量考核体系，促使各政府部门改进工作。着力提高政务公开的实效性，保障市民的知情权、参与权、表达权、监督权。建立健全工作责任制，加强督促检查和考核。注重发挥新闻媒体的舆论监督作用，加大对典型事例的曝光力度，纠正推诿扯皮、办事拖沓等行为。要进一步加强各级政府的基础工作，指导、支持基层组织做好服务。

　　(三)不断推进依法行政。

　　各级政府、各部门要深入贯彻国务院《全面推进依法行政实施纲要》，严格按照法定权限和程序行使权力、履行职责。深入基层，了解社情民意，完善听证会制度，进一步提高科学决策水平。大力推进政府管理创新和制度创新，提高依法行政能力和水平。建立行政问责制度，严肃查处失职渎职行为。认真贯彻监督法，全面执行人大及其常委会的决议，自觉接受监督。主动加强与人民政协的联系，认真听取各民主党派、工商联、无党派人士和人民团体的意见。坚持与人大代表、政协委员的联系制度，及时、高质量地办理议案、建议和提案。

　　(四)全面加强作风建设。

　　认真学习贯彻胡锦涛总书记在中纪委七次全会上的讲话精神，对当前党风廉政建设和反腐败斗争形势保持清醒认识，居安思危，建立健全教育、制度、监督并重的惩治和预防腐败体系，切实加强领导干部思想作风、学风、工作作风、领导作风、生活作风建设，倡导和树立“八个方面”的良好风气，常抓不懈，务求实效。坚持勤俭办一切事业，2007年将压缩政府部门经常性项目支出资金10%，强化对财政性资金使用、工程招投标的监察和审计，规范政府机关公务接待，杜绝铺张浪费。全体政府工作人员要心系群众，服务人民，切实纠正各种损害群众利益的不正之风，真正做到“为民、务实、清廉”。

　　各位代表，2007年下半年，党的十七大将在北京召开，这是全国人民政治生活中的头等大事。全市各级政府将切实负起责任，依靠全体市民的参与和支持，积极营造良好的社会环境，做好各项服务保障工作，为十七大的胜利召开做出首都人民应有的贡献。

　　2008年奥运会正迎面走来，世界的目光聚焦北京。让我们更加紧密地团结起来，在以胡锦涛同志为总书记的党中央正确领导下，肩负历史使命，不负人民重托，为全面实现“新北京、新奥运”战略构想，构建和谐社会首善之区而不懈奋斗！
